# Inherited and acquired vitamin B12 deficiencies: Which administration route to choose for supplementation?

**DOI:** 10.3389/fphar.2022.972468

**Published:** 2022-09-29

**Authors:** Ramyia Elangovan, Julien Baruteau

**Affiliations:** ^1^ Metabolic Medicine Department, Great Ormond Street Hospital for Children NHS Foundation Trust, London, United Kingdom; ^2^ Genetics and Genomic Medicine Department, Great Ormond Street Institute of Child Health, University College London, London, United Kingdom; ^3^ National Institute of Health Research Great Ormond Street Biomedical Research Centre, London, United Kingdom

**Keywords:** cobalamin, hydroxocobalamin, cyanocobalamin, vitamin B12, metabolic

## Abstract

Vitamin B12 or cobalamin deficiency is a commonly encountered clinical scenario and most clinicians will have familiarity prescribing Vitamin B12 to treat their patients. Despite the high prevalence of this condition, there is widespread heterogeneity regarding routes, schedules and dosages of vitamin B12 administration. In this review, we summarise the complex metabolic pathway of Vitamin B12, the inherited and acquired causes of Vitamin B12 deficiency and subsequently highlight the disparate international practice of prescribing Vitamin B12 replacement therapy. We describe the evidence base underpinning the novel sublingual, intranasal and subcutaneous modes of B12 replacement in comparison to intramuscular and oral routes, with their respective benefits for patient compliance and cost-saving.

## Introduction

In 1934, Whipple, Minot and Murphy were awarded the Nobel Prize for their game-changing discovery that feeding liver meat to patients affected by pernicious anaemia could act as a curative therapy for this previously invariably fatal condition (Whipple et al., 1920; Minot and Murphy, 1926). Subsequent decades of research, including Dorothy Hodgkin’s X-ray crystallography studies (Hodgkin et al., 1956), revealed that Vitamin B12 (otherwise known as cobalamin) was the important therapeutic compound curing these patients. This paved the way to prescribing B12 as a therapeutic compound to reverse cases of B12 deficiency.

Today, Vitamin B12 deficiency is a commonly encountered clinical diagnosis and most clinicians will have prescribed B12 replacement therapy for some of their patients. However, despite the widespread nature of this condition, there are no internationally standardised guidelines regarding dosage regimes, optimal formulation, frequency of administration of B12 therapy and monitoring of therapeutic effect, leading to significantly variable international practice (Andres and Serraj, 2012; Langan and Goodbred, 2017; Sobczyńska-Malefora et al., 2021).

In this review, we aim to summarise the complex pathway by which B12 is absorbed and metabolised and subsequently describe the inherited and acquired causes of B12 deficiency. We then summarise the disparate practice in B12 replacement internationally and highlight the more novel methods of B12 administration, including subcutaneous, intra-nasal and sublingual modes in comparison to intramuscular and oral routes.

### Vitamin B12 absorption and metabolism

**FIGURE 1:**
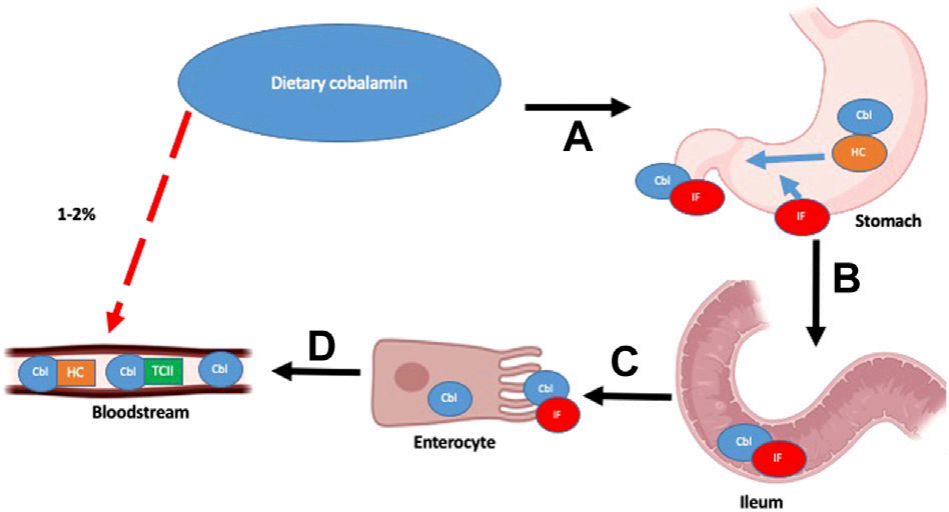
Vitamin B12 absorption pathways and main factors and organs involved: Receptor-mediated absorption processes shown as sequential steps **(A–D)** with black arrows and Passive diffusion process outlined with red, dashed arrow. Detailed description of pathway outlined in text. Adapted from “Human Anatomy, Digestive System,” by BioRender.com (2022). Retrieved from https://app.biorender.com/biorender-icons.https://app.biorender.com/biorender-icons

Vitamin B12 is a complex biomolecule that plays a vital part in key biochemical reactions. It is a water-soluble vitamin that is derived from the diet, particularly from eggs, red meat, and dairy. There are two distinct mechanisms by which B12 is absorbed: 1) passive diffusion across the mucous membranes of the gastrointestinal tract (Berlin et al., 1968) and 2) a receptor-mediated absorption process (Figure 1). Only an estimated 1%–2% of oral B12 can be passively absorbed and therefore, high dosages of oral B12 are required to provide therapeutic benefit in cases where the receptor-mediated process malfunctions (Withey et al., 1963).

In the receptor-mediated absorptive process, ingested B12 is initially bound to Haptocorrin (HC)/R-factor secreted by salivary glands. Free B12 is once again released following degradation of HC by pancreatic proteases in the duodenum. It subsequently binds to the intrinsic factor (IF), which is secreted by gastric parietal cells. This B12-IF complex reaches the terminal ileum, where it binds to heterodimeric receptors cubam composed of an outer protein *cubulin* and transmembrane protein *amnionless*, located on the surface of polarized epithelial enterocytes in the apical brush border (Fyfe et al., 2004). The complex is then endocytosed, and free B12 is released into the bloodstream.

In the bloodstream, free B12 may be bound by HC or transcobalamin (TC). B12 bound to HC is not taken up by cells and thus, is biologically inactive (Hunt et al., 2014). However, B12 bound to TC forms a complex known as holoTC, which is the biologically active form of B12, that is taken up into cells *via* endocytosis using a specific CD320 receptor (Quadros, 2010). Following endocytosis, the HoloTC complex is degraded in lysosomes and free B12 is released into the cytoplasm, *via* CblF (Rutsch et al., 2009) and CblJ (Coelho et al., 2012).

**FIGURE 2:**
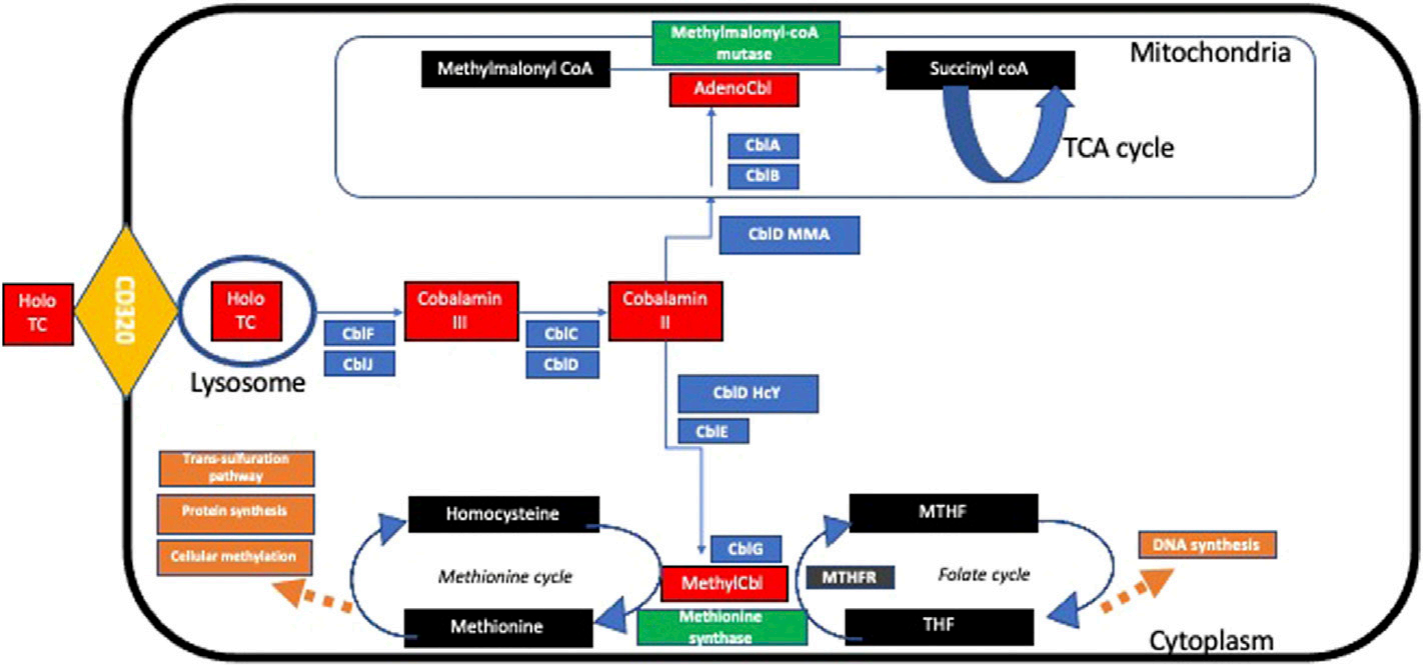
Summary of intracellular metabolism of transcobalamin. Detailed description of pathway found in text.

This free cytoplasmic B12 undergoes a series of intracellular transport and modifications to enable two core enzymatic reactions: 1) adenosyl-B12 acts as a co-factor for the enzyme methylmalonyl-coA mutase that catalyzes the conversion of methylmalonyl-coA to succinyl-coA, thereby enabling restoration of tricarboxylic acid (TCA) cycle intermediates and 2) methyl-B12 enables methionine synthase in the re-methylation of homocysteine to methionine using methyl-tetrahydrofolate (MTHF) as a methyl donor, thereby enabling the vital biochemical pathways of the folate cycle and methionine cycle, facilitating the downstream synthesis of important compounds like DNA and RNA (Figure 2).

### Causes of B12 deficiency and criteria for diagnosis

The absorption and metabolic pathway for B12 is highly complex, and defects in any part of this process can lead to therapeutic B12 replacement. The causes of B12 deficiency can be subdivided into congenital and acquired (Table 1).

**TABLE 1:** Causes of vitamin B12 deficiency, classified by cause.

Congential (with gene name, gene localisation, and OMIM number II)	Acquired
**Cobalamin-binding intrinsic factor deficiency (CBLIF)**	**Dietary**
• CBLIF,11q12.1,#261000	• Vegan/Vegetarian
	• Malnutrition
**Imerslund-Grasbeck Syndrome**	**Ileal resection**
• CUBN, 10p13,#261100	
• AMN,14q32.32,#618882	
**Cobalamin defects (CbIA-CbIG)**	**Medications**
• CblA-MMAB,4q31.21,#251100	• Metformin
• CblB-MMAB,12q24.11,#251110	• Proton pump inhibitor
• CblC-MMACHC,1p34.1,#277400	• Alcohol
• EpI-CblC-PRDX1,1 34.1,#176763	
• CbID-MMADHC,2q23.2,#277410	
• CbIE-MTRR,Sp15.31,#236270	
• CbIF-LMBRDI,6q13,#277380	
• CbIG-MTR,1q43,#250940	
• CbIJ-ABCD4,14q24.3,#614857	
• CbIX-HCFC1,Xq28,#309541	
• THAP11-THAP11.16q2.1,NA	
• ZnF143 deficiency-ZNF143,11p15.3,NA
**Transcobalamin II (TCII) deficiency**	**Gastric causes**
• TCN2,zzq12.2,#275350	• Pernicious anaemia
	• Gastrectomy
	• Gastric bypass
**Transcobalamin receptor (Haptocorrin) Mutation**	**Inflammatory Bowel Disease**
• TCN1,19p13.2,#613646	

Congenital causes are rare and are the result of genetic mutations in genes encoding proteins involved in B12 absorption, transport, and intracellular processing. These include 1) congenital pernicious anaemia, where there is a genetic defect in IF synthesis, leading to B12 malabsorption, 2) Imerslund-Gräsbeck Syndrome (IGS) caused by mutations in genes encoding *cubulin* and *amnionless*, 3) inherited HC or TCII deficiencies, and 4) intracellular cobalamin defects (CblA-CblG defects). Beyond mutations in the genes coding for these intracellular cobalamin proteins, there are also multiple mutations which affect the transcriptional regulation of MMACHC, leading to a similar phenotype of CblC deficiency (Watkins et al., 2017); In epi-CblC, an epimutation leads to a splicing variant in the adjacent PRDX1 gene causes transcriptional silencing of MMACHC (Guéant et al., 2018). As for CblX deficiency, mutations in the HCFC1 gene affect transcriptional expression of MMACHC (Yu et al., 2013). Finally, THAP11 deficiency (Quintana et al., 2017) and ZNF143 deficiency (Pupavac et al., 2016) also affect the transcriptional regulation of MMACHC. Acquired causes of B12 deficiency can be 1) dietary, especially in vegetarian and vegan populations, 2) autoimmune, as seen in pernicious anaemia, where there is destruction of IF-producing parietal cells mediated by autoantibodies, 3) iatrogenic secondary to disruption of the acid-base status of the stomach (e.g., with proton pump inhibitor use or metformin), and 4) caused by malfunction of the terminal ileum (e.g., inflammatory bowel diseases like Crohn’s disease, gastric bypass or surgical resection).

Regardless of the cause, the diagnosis of B12 deficiency is complex and can present at all ages with a wide spectrum of symptoms. Clinical presenting features of the condition include those relating to anaemia (e.g., pallor, weakness, and fatigue), gastro-intestinal symptoms (e.g., epigastric pain and glossitis), neurological symptoms (e.g., from numbness of extremities and impaired proprioception in subacute combined degeneration of the spinal cord to severe developmental delay, epilepsy, and coma in inherited intracellular cobalamin defects), psychiatric disturbances and neural tube defects in the newborn of a deficient mother (Molloy et al., 2009).

In cases which arouse clinical suspicion, there is no gold standard single test for the diagnosis of this condition (Solomon, 2005; Herrmann and Obeid, 2012), but multiple laboratory investigations can be performed. After all, serum B12 can be within the reference range even in cases of severe B12 deficiency (Devalia et al., 2014). This is also true where the therapeutic effect of B12 supplementation needs to be monitored as after all, as although low B12 levels may indicate deficiency, high B12 levels may not suggest sufficient treatment, especially in the context of persistent symptoms. Possible laboratory investigations which can be performed include serum B12 [i.e., <148 *p*mol/L cut-off in the UK (Devalia et al., 2014)] and holoTC levels. Confirmatory testing of homocysteine, methionine, and methylmalonic acid levels may also prove useful (Quadros, 2010; Carmel, 2011; Fedosov, 2012). It is important to note that these latter tests are not widely available and the diagnostic cut-off values differ between laboratories (Fedosov, 2012; Aparicio-Ugarriza et al., 2015). Whole exome sequencing or fibroblast complementation studies can be performed in suspected cases of genetic cobalamin defects to identify the disease-causing gene.

### Current guidelines for B12 replacement

Vitamin B12 is available in four different formulations—cyanocobalamin (CN-Cbl), hydroxocobalamin (OH-Cbl), methylcobalamin (methyl-Cbl), and adenosylcobalamin (adenosyl-Cbl)—depending on the R residual group attached to the core cobalamin molecule. These formulations have different affinities to key proteins and receptors which facilitate the generation of biologically active intracellular cobalamin and thus, differ in their rates of tissue retention and bioavailability (Paul and Brady, 2017). Additionally, the solvent body in which B12 is dissolved may also influence its overall absorption and bioavailability (Arendt and Nexø, 2011). Regardless of the form of B12 administered, all of them are ultimately reduced to the core cobalamin molecule within the cytoplasm before proceeding to downstream reactions outlined above (Obeid et al., 2015).

The formulation and dosage regimens of B12 used to treat Vitamin B12 deficiency vary widely depending on the aetiology of the deficiency as well as region of medical practice (Table 2). For inherited B12 deficiency, treatment is guided by the genetic condition. Although oral treatment has been trialled with some success (Gangarossa et al., 1996; Bor et al., 2008), the parenteral modality of B12 replacement [particularly intramuscular (IM)], has proven successful in the management of this cluster of rare diseases. In cobalamin-binding intrinsic factor deficiency, retrospective studies of patients with this rare condition have revealed that daily 1 mg intramuscular (IM) injections of OH-Cbl/CN-Cbl are effective and can be gradually spaced out depending on the metabolic and clinical response of patients, with patients eventually becoming stabilised on twice yearly 1 mg intramuscular (IM) injections of OH-Cbl/CN-Cbl (Abdallah et al., 2012). This same treatment regimen has also been used with success in Imerslund-Gräsbeck syndrome (Abdallah et al., 2012), although an alternative treatment regimen which has proven useful in this condition is 1 mg IM OH-Cbl daily for 10 days and then once a month for lifetime (Gräsbeck, 2006). As for cobalamin-related remethylation disorders (CblC or CblJ deficiency), daily 1 mg IM OH-Cbl has proven effective, with frequency of administration individually titrated according to metabolic response (Huemer et al., 2017). Another prospective review of 26 patients with late-onset CblC deficiency with neuropsychiatric manifestations demonstrated efficacy of a different treatment regimen (loading dose of 0.5–1 mg daily IM OH-Cbl or CN-Cbl for 4–8 weeks then maintenance dose of 0.5–1 mg weekly) in improving the metabolic and clinical parameters of these patients (Wang et al., 2019). As for transcobalamin II deficiency, an observational case series of 30 patients revealed that IM administration of 1 mg of OH-Cbl or CN-Cbl weekly for a lifetime appears to be a suitable treatment regimen (Trakadis et al., 2014).

**TABLE 2:** Formulation and dosage regimes for treatment of vitamin B12 deficiency, subdivided into congenital and acquired causes. Abbreviations: OH-Cbl, hydroxocobalamin; CN-Cbl, cyanocobalamin.

Congential	Acquired
**Cobalamin-binding intrinsic factor deficiency (CBLIF)**	**British National Formulary**
• 1 mg IM OH-CbI/CN-Cbl daily in severe pancytopenias until resolved and then spaced out according to metabolic parameters	• Inpatients without neurological involvement: 1 mg IM OH-Cbl 3 times a week for 2 weeks and then maintenance 1 mg IMOH-Cbl every 3 months
	• Inpatients with neurological involvement, Img IMOH-Cbl on alternate days until there is no clinical improvement and then maintenance 1 mg IM OH-Cbl every 2 months
• Eventually patients stabilized on twice yearly 1 mg CN-Cbl or OH-Cbl injections	• 50 μg low-dose CN-Cbl in asymptomatic, borderline cases
**Imerslund-Grasbeck Syndrome (IGS)**	**British Columbia Medical Association**
• 1 mg IM OH-CbI daily for 10 days and then once a month for lifetime OR	• For pernicious anemia or food-bound cobalamin malabsorption, 1 mg daily oral CN-Cbl.
• 1 mg IM OH-CbI/CN-CbI daily in severe pancytopenia until resolved and then spaced out according to metabolic parameters	• Inmost other cases a dose of 250 μg/day may be used
• Eventually patients stabilized on twice yearly 1 mg CN-Cbl or OH-Cbl injections with careful monitoring	• Reserve parenteral administration for those with neurological symptoms:
	• IM/SC 1 mg IM CN-Cbl OD for 1–5 days, followed by 1-2 mg OD oral CN-Cbl.
	• Ensure serum Cbl normalized after 4–6 months
**Cobalamin Defects**	**Netherlands**
• 1 mg IM OH-CbI daily, with frequency of administration individually titrated according to metabolic response OR	• Dutch Organization of General Practitioners 2014 viewpoint: Treat a cobalamin count below 148 pmol/L and clinical symptoms with 11 mg daily oral Cbl
• Loading dose of 0.5–1 mg daily IMOH-Cbl or CN-Cbl for 4–8 weeks then maintenance of 0.5–1 mg weekly OH-Cbl	• Dutch Healthcare institute Pharmacotherapeutic Compass: IM/SC loading dose of 10 injections of 1 mg OH-Cbl at intervals of ≥3 days; maintenance dose 1 mg once every 2 months or 300 ug/month, for lifelong supplementation if underlying cause not removed
	• In case of evident neurological disorders: 1 mg once or twice a week for 2 years.
**Transcobalamin II deficiency**	**France**
• IM administration of 1 mg of OH-Cbl or CN-Cbl weekly for lifetime	• CARE B12 research group recommendations:
	• Inpernicious anaemia: 1 mg oral CN-Cbl daily for life OR 1 mg IMCN-Cbl daily for 1 week then ‘once weekly for 1 month then monthly for life
	• 1–2 mg per day for at least 2–3 months in cases of moderate to severe neurological manifestations
	• In food-bound cobalamin malabsorption, Crohn's disease, malabsorption or dietary deficiency: 1 mg oral CN-Cbl daily for 1 month then 125 μ*g*–1 mg daily CN-Cbl until cause is corrected OR 1 mg IM CN-Cbl daily for 1 week then once weekly for 1 month then per month for 1–3 months until cause is corrected
	• 1 mg daily for 1–3 months in cases of severe neurological manifestations
**Haptocorrin deficiency**	**Australia**
• Treatment not indicated	• Royal Children’s Hospital (Melbourne), Immigrant Health Service—varying regimens
	• For infants with clinical deficiency: 250 μg–1 mg IM OH-Cbl (preferred) or CN-Cbl on alternate days for 1–2 weeks then 250 μg IM weekly, switch to oral when child is well
	• For older children with mild disease, 1 mg oral daily
	• Insub-clinical cases or with dietary deficiency, 50–200 jug oral daily

In acquired B12 deficiency, both oral and IM routes have been featured in guidelines to replenish this compound in deficient states. However, there does not appear to be internationally standardised practice in how this is achieved (Wentworth and Copland, 2018), and a non-exhaustive list of varying practice of a few key countries is summarised (Table 2). In Britain, the British National Formulary (BNF) recommends 1 mg IM OH-Cbl 3 times a week for 2 weeks and then maintenance 1 mg IM OH-Cbl every 3 months in patients without neurological involvement; as for patients with neurological improvement, 1 mg IM OH-Cbl on alternate days until there is no clinical improvement and then maintenance 1 mg IM OH-Cbl every 2 months is recommended (NICE, 2022). For asymptomatic, borderline cases, 50 μ g low-dose daily oral CN-Cbl is licensed (NICE, 2022).

In Canada, advice from the British Columbia Medical Association outlines that 1 mg daily oral CN-Cbl should be used for pernicious anemia or food-bound cobalamin malabsorption (FBCM) whereas in most other cases, a daily dose of 250 μ g may be used (British Columbia, 2013). Parenteral treatment is advised to be reserved for patients with neurological symptoms using a regimen as follows: IM/SC 1 mg IM CN-Cbl daily for 1–5 days, followed by 1–2 mg daily oral CN-Cbl, with biochemical testing to ensure serum Cbl normalized after 4–6 months (British Columbia, 2013).

In Netherlands, controversy arose when the Dutch Organization of General Practitioners published a 2014 viewpoint that 1 mg daily oral OH-Cbl can be utilised as treatment in cases with a cobalamin count below 148 pmol/L and clinical symptoms, with the Dutch B12 Society disputing the efficacy of this and insisting that the advice from the *Dutch Healthcare Institute Pharmacotherapeutic Compass* should be followed instead (IM/SC starting dose of 10 injections of 1 mg OH-Cbl at intervals of at least 3 days; maintenance dose once every 2 months or 300 μ g once a month, but In cases of evident neurological disorders: 1 mg once or twice a week for 2 years) (Author Anonymous, 2022; College voor Zorgverzekeringen, 2011).

In France, the Strasbourg-based *CARE B12* research group recommends differing treatment regimens for pernicious anaemia and other causes of B12 deficiency (Andrès et al., 2018); In pernicious anaemia, the treatment regimens of either 1 mg daily lifelong oral CN-Cbl or 1 mg IM CN-Cbl daily for 1 week then once weekly for 1 month then monthly for life are recommended. However, in cases with moderate to severe neurological manifestations, higher doses of 1–2 mg per day for at least 2–3 months are recommended. In food-bound cobalamin malabsorption, Crohn’s disease, malabsorption, or dietary deficiency, either 1 mg daily oral CN-Cbl daily for 1 month and then 0.125–1 mg daily CN-Cbl until cause is corrected or 1 mg IM daily CN-Cbl for 1 week then once weekly for 1 month then per month for 1–3 months until cause is corrected are recommended. A higher dosage regimen of 1 mg daily for 1–3 months is recommended in cases of severe neurological manifestations (Andrès et al., 2018).

In Australia, the Immigrant Health Service of the Royal Children’s Hospital of Melbourne recommends varying treatment regimens based on age of the patient (Immigrant Health Service, 2013); for infants with neurological involvement or macrocytic anaemia, a 250 μ g-1mg IM OH-Cbl (preferred) or CN-Cbl daily is recommended on alternate days for 1–2 weeks then 250 μ g weekly injections until clinically improved, before switching to oral replacement treatment. In older children with mild disease, a 1 mg daily oral treatment regimen is suggested (Immigrant Health Service, 2013).

The different recommended doses, formulations and treatment schedules both between and within these countries highlight the important disparity in international clinical practice for the treatment of this heterogenous condition. Nevertheless, the IM route of either OH-Cbl or CN-Cbl remains more established and widely recommended for treatment of B12 deficiency.

### Oral B12

There is growing popularity for the oral route of B12 replacement, and as outlined above, it is recommended in various countries as a potential treatment modality for B12 deficiency. In fact, it has proven to be the preferred mode of treatment in numerous countries including Sweden, Norway, and Canada (Nilsson et al., 2005). This is largely in view of the high cost savings, ease of administration and reduced pain and injection-associated injury (Wentworth and Copland, 2018).

Three key trials have been comparing IM and oral administration modalities for B12 replacement in small patient groups with varying causes of B12 deficiency. Doses and frequency of administration were variable but ranged anywhere from 0.5 to 2 mg (Kuzminski et al., 1998; Bolaman et al., 2003; Sanz-Cuesta et al., 2020). A recent Cochrane review of these trials, involving 153 patients, found comparable serum B12 levels in patients taking either route (Wang et al., 2018). It is important to highlight that none of these trials looked at the clinical signs and symptoms of these patients nor outcomes relating to their quality of life. Furthermore, observation of patient compliance to daily oral B12 intake is difficult to assess and might affect patient biochemistry and outcomes long-term (Stabler et al., 2013). Finally, none of these studies included intention-to-treat analysis, blinding or sample size calculation, which may have introduced bias in their statistical analysis (Vidal-Alaball et al., 2004; Wentworth and Copland, 2018). Thus, IM administration remains the preferred route of B12 replacement in cases of known malabsorption or severe deficiency.

### Sublingual B12

The sublingual route can also be considered for replacement therapy in B12 deficiency. With sublingual administration, the complex B12 absorption process can be bypassed and B12 can directly reach the bloodstream *via* the sublingual veins. With the relative ease and painless nature of administration, it is especially considered in the paediatric population. Early evidence for the potential efficacy of this route was published in 1999, where 18 patients with B12 deficiency treated exclusively with 7–12 days of 2 mg of sublingual cobalamin daily had 4 times higher B12 levels compared to pre-treatment, reaching therapeutic ranges (Delpre et al., 1999). A further study demonstrated that a dose as small as 0.35 mg once weekly of CN-Cbl over 12 weeks may be sufficient at normalising serum B12 levels (Del Bo et al., 2019).

There have also been further studies comparing treatment outcomes from the sublingual route to the more established IM and oral routes. Sharabi et al. (2003) compared the efficacy of 0.5 mg daily doses of CN-Cbl in either sublingual or oral formulation for 30 subjects with low serum concentrations of B12 and found that B12 levels normalised in 4 weeks for both treatment groups, with no significant difference between the groups. A further Israeli study from 2019 performed a retrospective case note review of >4,000 patients with B12 deficiency treated with sublingual or IM B12 (but without detailing dosages and schedules of administration included in the study) and showed that the mean difference in serum B12 levels before and after treatment was significantly higher in the sublingual than IM group (Bensky et al., 2019).

Tuğba-Kartal and Çağla-Mutlu (2020) also demonstrated in a retrospective review of 129 paediatric patients that a treatment regimen of sublingual B12 (both CN-Cbl and methyl-Cbl) at 1 mg once daily for 7 days then every other day for 3 weeks was as effective as the same dose schedule of IM B12 at normalising serum B12 levels. Finally, a recent 2021 study performed in a paediatric population (0–3 years) in Turkey found that a sublingual methyl-Cbl treatment regimen, comprising 1 mg daily replacement for a week followed by every other day for 2 weeks, 2 days/week for 2 weeks and once a week for 3 months was as effective as similar dose schedules of IM and oral CN-Cbl at normalising B12 levels (Orhan Kiliç et al., 2021).

Although these studies report promising results regarding the sublingual route, it is important to note that almost all of them only include patients with mild, subclinical B12 deficiency, making it difficult to extrapolate their outcomes for patients with severe deficiency or malabsorptive states. These studies often took place for a few week- or month-long period, making it difficult to understand the longer-term potential of sublingual B12 at maintaining serum B12 levels. Sublingual B12 was administered in “laboratory” conditions, where there was no dose omission, making it difficult to extrapolate “real-world” effects, where patient compliance may not be as good. Furthermore, they only look at serum B12 as a biomarker of B12 deficiency and do not consider methylmalonic acid, homocysteine, methionine, haematological parameters, or clinical symptomatology in their assessment of therapeutic effect. Therefore, it remains difficult to recommend with confidence this route of administration for all patient populations.

### Intranasal B12

Similar to sublingual B12, intranasal B12 can also bypass the complex B12 absorption process and directly reach the bloodstream. With the ease of administration, it also possesses the same advantages as sublingual therapy for patients and has been shown to be a preferred treatment modality to the IM route (Suzuki et al., 2006). However, evidence of efficacy of this route compared to more established treatment routes remains limited. One study from 1997 recruited six patients with plasma B12 levels of <200 ng/L and administered a 1.5 mg intranasal dose of OH-Cbl at day 0, 14, and 21, subsequently measuring plasma B12 concentrations at timepoints between 1 h and 35 days following administration. This study showed an 8 times increase in the mean cobalamin concentration sustained 1 week following the final dose (Slot et al., 1997). A further small study done recently on 10 paediatric patients similarly found that intranasal OH-Cbl normalised serum B12 levels in deficient patients (Estourgie-van Burk et al., 2020).

### Subcutaneous B12

Subcutaneous B12 is occasionally used, especially in patients with inborn errors of cobalamin metabolism, who need high pharmacological doses of B12 to maintain their B12-dependent metabolic pathways and related biochemistry within normal range. Studies looking at the efficacy of the subcutaneous route at replenishing B12 levels in depleted states are limited. Serum and liver vitamin B12 concentrations were found to be significantly elevated for 24 days in lambs treated with a single 2 mg subcutaneous vitamin B12 injection, compared to untreated controls (Grace et al., 1998).

There have also been a few case series published reporting the general efficacy of the subcutaneous route at normalising plasma B12 levels; Lotz-Havla et al. (2021) described the subcutaneous administration of OH-Cbl in four paediatric patients (two patients with CblC deficiency, one patient with CblG deficiency and one patient with CblE deficiency). Total homocysteine levels remained within the target range with this alternative route of administration (vis-à-vis IM B12 which the patients had previously been established on) and was described as a more pain-free and acceptable treatment option for all patients studied. A further case report outlined a subcutaneous port system inserted to deliver daily OH-Cbl for a patient with CblA deficiency—the patient in this case, who previously had been stable on IM B12 injections, had normal methylmalonic acid levels throughout the study period (Maines et al., 2016).

It is critical to note that a rigorous comparison of subcutaneous to IM and oral treatment modalities have not been published yet. This makes it difficult to suggest it as an equal alternative to more established treatment modalities for B12 deficiency.

### Potential cost savings

There are numerous cost savings generated when switching from IM B12 to alternative treatment routes. A previous cost minimization analysis from UK prescribing patterns and costs, revealed that switching patients to oral B12 from the IM route could lead to 50% cost savings (Vidal-Alaball et al., 2006). Most savings could come from the reduction of resources associated with home visits. A further study performed in Canada estimated that switching patients established on IM B12 to the oral route could save the single province of Ontario between 1.4 and 9 million US Dollars over a 5-year period (Van Walraven et al., 2001). These cost savings include costs for room rental/upkeep, needle disposal as well as remuneration for attendant health professionals. We hypothesize that consideration of sublingual/intranasal B12 will also lead to similar savings, due to similar ease of administration as oral B12.

## Conclusion

B12 deficiency is a common diagnosis in clinical practice. Causes are numerous and the diagnostic process can be complex. The therapeutic management based on B12 supplementation remains heterogeneous both between and within countries on the treatment routes, schedule, and dosage regimens. If oral and intramuscular injections have been proposed historically, alternative treatment routes have been recently proposed with sublingual, intranasal and subcutaneous administrations, which are more patient-friendly than painful intramuscular injections. This is particularly true for paediatric patients. The evidence base requires appropriate clinical trials, with additional endpoints including clinical symptoms of patients and additional biochemical parameters beyond plasma B12 levels alone before these alternative routes can be recommended with confidence in clinical guidelines. It is essential that clinical trials can be developed to effectively compare these routes of administration and that clinicians can share their experiences with these modalities of B12 administration so that they can be popularised and considered as viable alternatives for patients. This need becomes even more pressing in view of the potential cost-savings these alternatives will provide for financially stressed public health services. Nevertheless, in cases of severe deficiency with neurological sequelae, we suggest that IM B12 is used in the first instance to replenish body stores with the treatment regimen, including dosage and formulation, optimised to keep the patient free of symptoms and tailored to the metabolic response of not only plasma B12 levels, but also confirmatory testing of homocysteine, methionine, and methylmalonic acid levels.
